# Editorial: Applying robotics and AI in pandemics (COVID-19): Detection, diagnosis and delivery

**DOI:** 10.3389/frobt.2022.1039273

**Published:** 2022-09-26

**Authors:** Bin Fang, Hang Su, John Oyekan

**Affiliations:** ^1^ Department of Computer Science and Technology, Tsinghua University, Beijing, China; ^2^ The Department of Automatic Control and Systems Engineering, The University of Sheffield, Sheffield, United Kingdom

**Keywords:** autonomous agents, pandemics (COVID-19), detection, infection control, delivery

The COVID-19 pandemic highlighted the fragility of the human response to pandemics on a global scale. First, the airborne transmission of the virus has led to a significant increase in infections. Weak medical systems ineffectively isolate the source of the disease. Second, close contact increases the probability of cross-infection and puts understaffed health workers at risk. In addition, the lack of detection devices limits tracking the health status of people and diagnosing cases in real time. Finally, the strict protection policy has led to a sharp decline in transportation efficiency and shortages of supplies. These factors make COVID-19 have an unprecedented impact on human society.

After 2 years of fighting COVID-19, humans are lessening the virus’s influence with robotics and artificial intelligence (AI). Robots make up for the lack of professionals. It has unique advantages in improving work efficiency and blocking virus transmission. For example, temperature detection robots have tracked health status in public places. Telemedicine robots can reduce close interactions between caregivers and infected patients. The COVID-19 throat swab intelligent sampling robot has achieved a single sampling success rate of more than 95%, improving the standardization of biological sample Research Topic and ensuring specimen quality. In this Research Topic, we aim to collect current research around robotics and AI in the pandemic to overcome the challenges preventing wide-scale robotic deployment. Our purpose is to address the feasibility and impact of robotics in COVID-19 diagnosis and detection.

This Research Topic contains nine research articles that present the newest research achievements of robotic applications: four papers focus on detection methods and devices; three papers are on the diagnosis of symptoms or viruses; and the remaining two papers study the delivery of healthcare resources.

The first paper (Zhou et al.) proposes a cough recognition network (CRN) based on the CNN model and a Mel-spectrogram. CRN can achieve excellent performance in cough recognition compared to other methods. The results of generalization tests show that CRN adapts to cough monitoring in complex scenes in daily life. It is expected to be a potential solution for disease management during the COVID-19 pandemic and to reduce health care workers’ exposure.

The second paper (Jumlongku) presents the effectiveness of a new method of using robot-assisted surgery to cut the skull of a cadaver. Compared with the oscillating saw method, the noise level is reduced. It also limits the release of aerosol particles to levels similar to those of ordinary autopsy procedures. This method reduces fine infectious dust particles, especially during the COVID-19 pandemic.

The third paper (Al-Zogbi et al.) proposes an autonomous robotic solution enabling POCUS scanning of COVID-19 patients’ lungs for diagnosis and staging. Under a CT scan, an algorithm is developed to estimate the optimal position and orientation of a US probe on a patient to image target points in the lungs. Without CT data, a deep learning algorithm can predict the 3D landmark positions of a ribcage given a torso surface model. The results demonstrate the preliminary feasibility of the system and its potential for offering a solution to help mitigate the spread of COVID-19 in vulnerable environments.

The fourth paper (McGinn et al.) presents the design of a robotic UVGI platform. It can be deployed alongside human workers and operate autonomously within cramped rooms. The results show that UVGI can effectively inactivate germs on commonly touched surfaces in radiology suites. Despite the short irradiation period, UVGI can inactivate microbes with more complex cell structures, thus indicating a high likelihood of effectiveness against COVID-19.

The fifth paper (Jumlongkul and Chutivongse) proposes an AMBU ventilator consisting of an automated AMBU bag ventilator, a negative pressure head box, and a transporting capsule. It allows the initial setting of the preliminary flow rate, rhythm, and volume of oxygen during the intubation period to protect medical colleagues from potential erosol infection. It is expected to block airborne transmission of COVID-19 viruses during the procedure.

The sixth paper (Akbari et al.) proposes a robot-assisted system that automatically scans tissue to enable isolation between patients and sonographers during the COVID-19 pandemic. This system automatically scans the tissue using a dexterous robot arm that holds the US probe and assesses the quality of the acquired US images in real time. According to the quality assessment algorithm, this system successfully maintains US image quality and is fast enough for use in a robotic control loop.

The seventh paper (Cheng and Tavakoli) reviewed applications requiring physiological organ motion compensation in the medical telerobotic system. Physical distancing restrictions cause significant effects on the delivery of physical healthcare procedures worldwide to limit the spread and transmission of the novel coronavirus. Medical telerobotic systems can play a positive role in the provision of telemedicine. This paper focuses on control-theoretic approaches to outline possible future directions of telerobotic systems in COVID-19 healthcare.

The eighth paper (Grimshaw and Oyekan) proposes a cable-driven parallel manipulator (CDPM) to balance an unstable load, a ball plate system. It consists of eight cables attached to the end effector plate. The hardware includes a reinforcement-learning trained neural network controller and nested PID controller to output the desired platform response. During the COVID-19 pandemic, this robotic device can reduce workers’ exposure.

The ninth paper (Cheung et al.) surveys the available army doctrine of healthcare missions. The proposed framework adopts metrics of spacing error, separation distance, and string stability to compare the performance of autonomous convoys. According to hierarchical decision-making, this paper argues for using nonlinear battlefield techniques for delivering healthcare logistics to remote pandemic outbreak areas.

These nine articles are strictly selected based on many submissions. We hope that the discussion of recent advances in this area can promote an understanding of how robotics and AI can be used to diagnose and detect COVID-19 and provide a framework for how we can integrate smart systems to reduce pressure on healthcare systems during current and future pandemics.

